# Preparation of Calcium- and Magnesium-Fortified Potato Starches with Altered Pasting Properties 

**DOI:** 10.3390/molecules190914556

**Published:** 2014-09-15

**Authors:** Takahiro Noda, Shigenobu Takigawa, Chie Matsuura-Endo, Koji Ishiguro, Koichi Nagasawa, Masahiro Jinno

**Affiliations:** 1 NARO Hokkaido Agricultural Research Center, Shinsei, Memuro, Kasai, Hokkaido 082-0081, Japan; E-Mails: takigawa@affrc.go.jp (S.T.); mechie@affrc.go.jp (C.M.-E.); kuro@affrc.go.jp (K.I.); nagako@affrc.go.jp (K.N.); 2 Jinno Potato Starch Co., Sarabetsu, Kasai, Hokkaido 089-1542, Japan; E-Mail: jinno@netbeet.ne.jp

**Keywords:** calcium, magnesium, potato starch, pasting properties

## Abstract

Calcium- and magnesium-fortified potato starches were prepared by immersion in various concentrations of CaCl_2_ and MgCl_2_ aqueous solutions, respectively. The pasting properties, *i.e.*, peak viscosity and breakdown, of all the starches obtained above were analyzed using a Rapid Visco Analyzer. Furthermore, the gelatinization properties and *in vitro* digestibility of the representative calcium- and magnesium-fortified starches were tested. The maximum calcium content of the fortified potato starches was as high as 686 ppm with the addition of a high-concentration CaCl_2_ solution, while the calcium content of the control potato starch was 99 ppm. The magnesium content increased from 89 to 421 ppm by treatment of the potato starch with an MgCl_2_ solution. Markedly lower values of peak viscosity and breakdown were observed in calcium- and magnesium-fortified potato starches than in the control potato starch. However, the gelatinization temperature and enthalpy as well as resistant starch content of calcium- and magnesium-fortified potato starches were similar to those of the control potato starch. It is concluded that potato starches with altered pasting properties can be easily manufactured by the use of solutions containing high levels of calcium and magnesium.

## 1. Introduction

Potato starch has been manufactured in local factories in Hokkaido, the northernmost island of Japan, and has been widely incorporated into many foods including fish paste products, noodles, and shrimp crackers. Small amounts of phosphate monoesters exist in native starch molecules. It has been established that potato starch contains high starch-bound phosphate compared with starches isolated from other plants [1,2,3,4,5,6]. In addition, potato starch naturally contains metal cations that are attached to the phosphate ester groups by ion forces [7,8,9,10]. There has been great interest in the health benefits of the intake of calcium and magnesium because they can help to reduce the risk of lifestyle-related diseases, such as type 2 diabetes [11,12] and osteoporosis [13]. It is important for food producers to seek food materials that would be adequate carriers of calcium and magnesium, important mineral elements in which humans are often deficient. Potato starch is suitable for application as a carrier of calcium and magnesium. The cation in the starch extracted from potatoes with distilled water in the laboratory is mainly potassium [7,10]. In starch extracted with tap water, some of the potassium is displaced by calcium, magnesium and other cations through an ion-exchange process [8]. As the tap water used for potato starch extraction in Hokkaido has extremely small amounts of calcium, magnesium, and other cations, the degree of the displacement is small. Thus, potato starches produced in local factories in Hokkaido generally have low concentrations of calcium (<200 ppm) and magnesium (<150 ppm) [7,9,10]. Pasting properties are important quality parameters of starches. Amylograph and Rapid Visco-Analyzer (RVA) tests, in which viscosity is monitored against time during a standard heating-cooling cycle with continuous stirring, are frequently used for measuring starch pasting properties [14,15,16]. Starch granules swell during initial heating, resulting in an increase in viscosity. Thus, starch exhibits a definite peak viscosity on Amylograph and RVA charts. Stirring causes swollen granules to disintegrate; as a result, viscosity subsequently decreases. Breakdown is regarded as a measure of the degree of the disintegration of granules or paste stability. The presence of phosphates in starch molecules enhances the starch pastes after gelatinization; hence, starch with higher phosphorus content exhibits a higher peak viscosity [2,5,6,8,17]. In addition, a level of divalent cations, such as calcium and magnesium, appeared to have a substantial impact on the pasting properties of starch, presumably by ionically cross-linking starch phosphate esters. In an early study of calcium-fortified potato starch, a higher calcium content was associated with a lower peak viscosity and breakdown, implying that calcium-fortified potato starch showed good viscosity stability [7]. Recently, Fortuna *et al.* [18] also reported definite decreases in peak viscosity and breakdown due to the magnesium-fortification of potato starch. Foods with desirable textural characteristics may be manufactured using calcium- and magnesium-fortified potato starches with unique pasting properties. Therefore, the fortification of potato starch with calcium and magnesium has brought interest with an increasing awareness of the importance of alternate pasting properties besides higher intake of calcium and magnesium. However, to the best of our knowledge, there are very few articles that have investigated effective and practical methods for preparing calcium- and magnesium-fortified potato starches.

The objective of this study was to prepare calcium- and magnesium-fortified potato starches by immersions of potato starch produced in a local factory in Hokkaido in aqueous solutions of CaCl_2_ and MgCl_2_ of various concentrations and to measure the pasting properties of all of the starches obtained above using RVA. We assessed the effects of calcium and magnesium enrichment on other starch properties. For this purpose, we analyzed the gelatinization properties of the representative calcium- and magnesium-fortified starches using differential scanning calorimetry (DSC) as well as the *in vitro* digestibility of the starches.

## 2. Results and Discussion

### 2.1. Mineral Content of the Control Potato Starch

**Figure 1:**
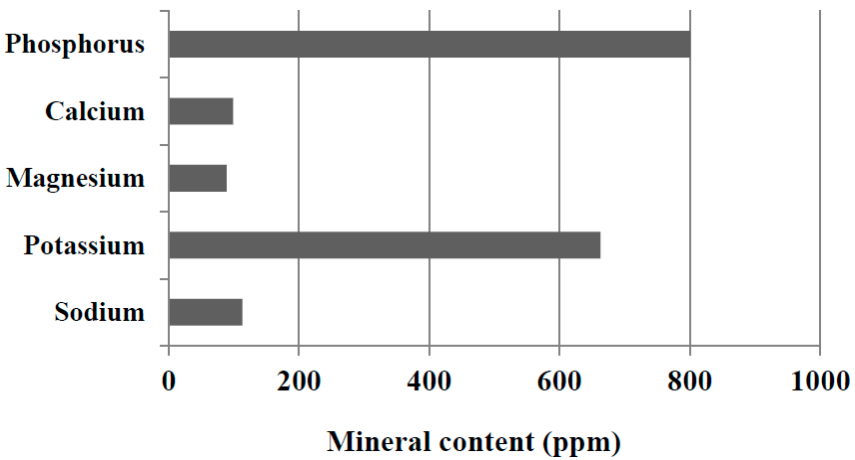
The content of phosphorus, calcium, magnesium, sodium, and potassium of the control potato starch.

The content of five minerals—phosphorus, calcium, magnesium, sodium, and potassium—of the control potato starch is shown in Figure 1. The phosphorus content of the control potato starch was as high as 801 ppm, which was in accordance with the content reported in the previous literature from our laboratory [6,9,10,19,20]. Namely, the variations in the phosphorus content of the potato starches manufactured at local factories in Hokkaido were within ranges of 416 to 1050 ppm [6], 715 to 1128 ppm [9], 541 to 1125 ppm [10], 644 to 1128 ppm [19], and 416 to 947 ppm [20] in this literature. The potassium content of the control potato starch was high (663 ppm), whereas the starch contained markedly lower calcium (99 ppm), magnesium (89 ppm), and sodium (113 ppm). In accordance with the previous reports on levels of cations in factory-made potato starches [7,9,10], the potassium content was generally higher (500 to 1000 ppm) than the content of calcium (50 to 200 ppm), magnesium (80 to 150 ppm), and sodium (30 to 100 ppm). 

### 2.2. Preparation of Calcium- and Magnesium-Fortified Potato Starches

**Figure 2:**
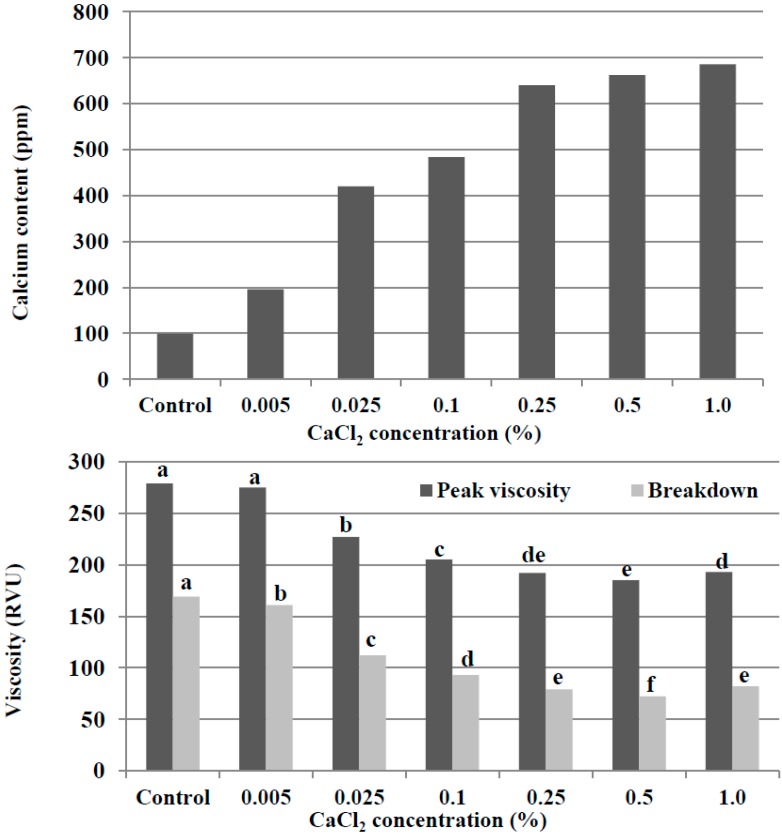
The calcium content and RVA pasting properties of the potato starches treated with CaCl_2_ aqueous solution. For each RVA parameter (peak viscosity or breakdown), bars labeled with the same letter are not significantly different (*p* < 0.05).

Due to the fact that the control potato starch is lacking in calcium and magnesium, we attempted to prepare calcium- and magnesium-fortified potato starches from the control potato starch and analyzed the pasting properties of these potato starches using RVA. First, 100 g of the control potato starch was treated with different amounts of calcium using 300 mL of CaCl_2_ aqueous solution. As can be seen in Figure 2, the calcium content of the treated potato starches increased drastically with the increase of the CaCl_2_ concentration up to 0.1%. In contrast, varying CaCl_2_ concentrations, from 0.25% to 1% resulted in an almost constant calcium content in the treated starches (640 to 686 ppm). In the RVA test, peak viscosity and breakdown were measured, and the values of peak viscosity and breakdown of the control potato starch were 279 and 169 RVU, respectively. In response to the results of the calcium content of the treated potato starches, peak viscosity and breakdown decreased markedly with the enhancement of CaCl_2_ concentration up to 0.1%. Similar values of peak viscosity (185 to 193 RVU) and breakdown (72 to 82 RVU) of the treated potato starches were observed with the addition of 0.25% to 1% CaCl_2_ aqueous solutions. 

**Figure 3:**
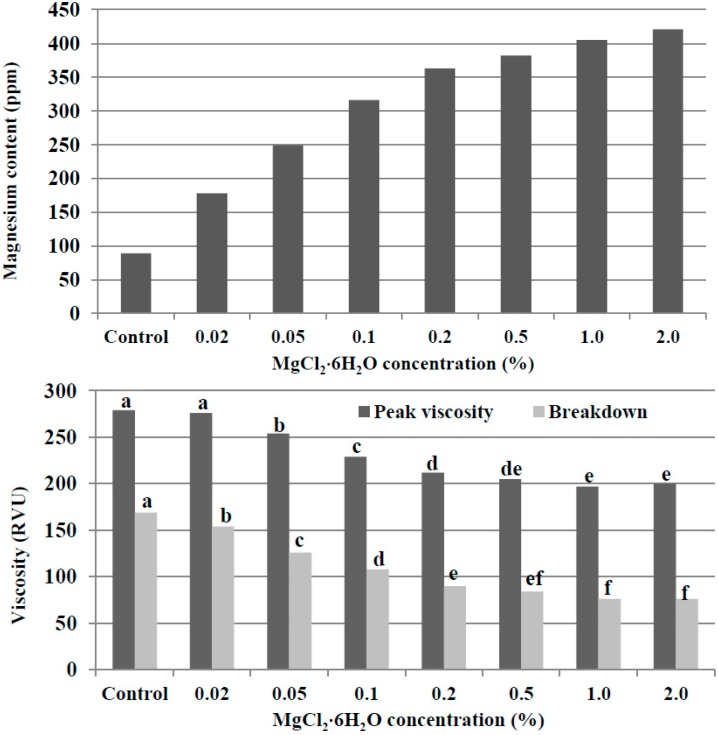
The magnesium content and RVA pasting properties of the potato starches treated with MgCl_2_ aqueous solution. For each RVA parameter (peak viscosity or breakdown), bars labeled with the same letter are not significantly different (*p* < 0.05).

Next, 100 g of the control potato starch was treated with different amounts of magnesium by use of 300 mL of an MgCl_2_ aqueous solution. As shown in Figure 3, a manifest increase in the magnesium content of the treated starch occurred with the increase in MgCl_2_·6H_2_O concentrations up to 0.2%. Above a 0.5% MgCl_2_·6H_2_O concentration, the magnesium content of the treated starch tended to be relatively constant (382 to 421 ppm). Similar to the trend of the magnesium content of the treated potato starches, an increased concentration of MgCl_2_·6H_2_O, up to 0.2%, led to a definite decrease in peak viscosity and breakdown. There were no significant differences in peak viscosity (197 to 205 RVU) and breakdown (76 to 84 RVU) of the treated potato starches at treatment at concentrations of 0.5% to 2% MgCl_2_·6H_2_O. 

From the results obtained here, we set up standard conditions for the preparation of calcium- and magnesium-fortified potato starches as follows: (i) immersion in 0.5% CaCl_2_ concentration for the calcium-fortified starch; and (ii) immersion in 1% MgCl_2_·6H_2_O concentration for the magnesium-fortified starch. Thus, we established an effective way to prepare calcium- and magnesium-fortified potato starches with drastically decreased values of peak viscosity and breakdown.

### 2.3. Properties of Calcium- and Magnesium-Fortified Potato Starches

**Figure 4:**
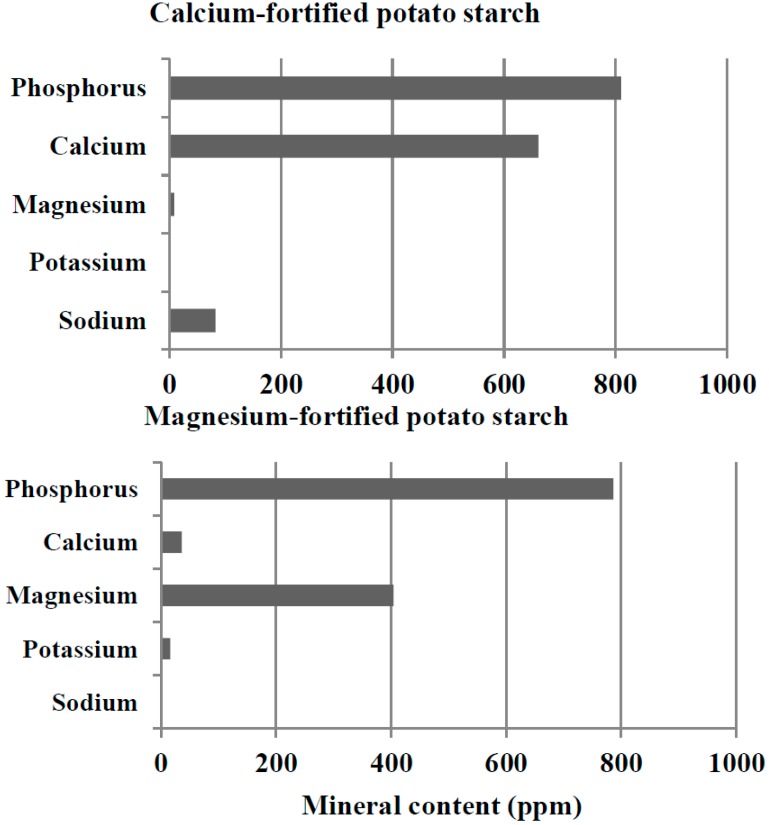
The content of phosphorus, calcium, magnesium, sodium, and potassium of the calcium- and magnesium-fortified potato starches.

We attempted to examine the properties of calcium- and magnesium-fortified potato starches obtained under the standard conditions mentioned above and to compare them with those of the control potato starch. First, we compared the mineral composition of the calcium- and magnesium-fortified potato starches with that of the control potato starch. As shown in Figure 1 and Figure 4, the phosphorus content of calcium- and magnesium-fortified potato starches was similar to that of control potato starch. The cation content of the calcium- and magnesium-fortified potato starches is also presented in Figure 2. The calcium-fortified potato starch had small amounts of sodium (82 ppm) and magnesium (8 ppm). In addition, potassium, which is the main cation in the control potato starch, was not detected in the calcium-fortified potato starch. Similarly, very small amounts (<4 ppm) of sodium, potassium, and calcium were found in the magnesium-fortified potato starch. It was proved that potassium in the control potato starch was replaced by calcium and magnesium during immersions in CaCl_2_ and MgCl_2_ aqueous solutions, respectively.

Thermal properties were measured by DSC, and the results of the calcium- and magnesium-fortified potato starches as well as the control potato starch are presented in Table 1. DSC parameters recorded were onset temperature (To), peak temperature (Tp), and enthalpy (∆H) for gelatinization. The To and Tp of all potato starches examined ranged between 62.6 and 63.1 °C and between 66.7 and 67.2 °C, respectively. To and Tp were significantly but slightly higher in the calcium- and the magnesium-fortified potato starches than in the control potato starch. The calcium and magnesium-fortified potato starches exhibited significantly but slightly lower ∆H (15.9 to 16.1 J/g) than the control potato starch (16.7 J/g). The data obtained here suggest that fortification of potato starch with calcium and magnesium did not have large impacts on the DSC thermal parameters, To, Tp, and ∆H.

To determine resistant starch (RS) content, each raw starch was treated with pancreatic α-amylase and amyloglucosidase for 16 h at 37 °C. As can be clearly seen from Table 1, all potato starches examined had extremely high content of RS content (93.0% to 95.5%) and no significant difference in RS content was observed among these starches. Thus, it was demonstrated that calcium- and magnesium-fortification of the control potato starch had no influence on RS content.

**Table 1:** DSC gelatinization properties and RS content of the calcium- and magnesium-fortified potato starches.

		Control	Calcium-Fortified Potato Starch	Magnesium-Fortified Potato Starch
DSC parameters	To (°C )	62.6 ± 0.2 ^c^	63.1 ± 0.1 ^a^	62.9 ± 0.2 ^b^
	Tp (°C)	66.7 ± 0.1 ^b^	67.2 ± 0.1 ^a^	67.1 ± 0.1 ^a^
	∆H (J/g)	16.7 ± 0.5 ^a^	15.9 ± 0.1 ^b^	16.1 ± 0.2 ^b^
RS content	(%)	95.1 ± 2.7 ^a^	93.0 ± 0.7 ^a^	95.5 ± 2.2 ^a^

The data are averages ± standard deviation of three and four determinations for DSC parameters and RS content, respectively. Values with the same letter in the same row are not significantly different (*p* < 0.05).

The dietary roles of calcium and magnesium for health promotion and disease prevention have been widely accepted. As starch is widely used in food production systems and contributes largely to the textural properties of the foods, the fortification of starches with calcium and magnesium would be a promising technology. Until now, modified starches, which are highly capable of binding metal elements, were used as carriers of calcium and magnesium, important mineral elements. Calcium fortification was reported for hydroxypropyl starches [21,22], and the magnesium fortification for oxidized starches [23]. Owing to the presence of naturally occurring phosphates, potato starch is able to attach metal cations, such as calcium, magnesium, potassium, and sodium, to polysaccharide chains to form a metal-starch complex. Potato starch would be an adequate carrier of calcium and magnesium without any chemical modification. However, the potato starch manufactured at a local factory in Hokkaido contains a large amount of potassium and small amounts of calcium and magnesium. Thus, it is important to manufacture calcium- and magnesium-fortified potato starches to enhance the nutritional value of foods that contain potato starch. Calcium-fortified potato starches (calcium content 516 to 784 ppm) were prepared earlier by Kainuma *et al.* [7]; however, their method included treating the control potato starch sequentially with 0.05 mol/L HCl, distilled water, saturated Ca(OH)_2_ solution, and distilled water, which is time consuming and not necessarily suitable for the industrial production of calcium-fortified potato starches. Wiesenborn *et al.* [8] suggested that the potato starch extracted in tap water, presumably containing a high level of calcium, was relatively high in calcium (207 ppm). According to a recent study by Fortuna *et al.* [18], treatment of the control potato starch with a mixture of 1% (w/w) MgCl_2_ and saturated Mg(OH)_2_ solution in a volume ratio of 1:1 resulted in an increased magnesium content (322.6 ppm). However, Fortuna *et al.* [18] did not provide data for the phosphorus content of the potato starches examined. Furthermore, the condition for the reaction of magnesium fortification was not investigated in detail. 

In this article, we have described a convenient and practical method for preparing calcium- and magnesium-fortified potato starches from factory-made potato starch by immersions in various concentrations of CaCl_2_ and MgCl_2_ aqueous solutions, respectively. The calcium- and magnesium-fortified potato starches obtained in this study had more than six times higher calcium content (maximum 686 ppm) and over four times higher magnesium content (maximum 421 ppm), respectively, than did the control potato starch. On a molar basis, the ratios of calcium and magnesium to phosphorus were approximately two-thirds for calcium- and magnesium-fortified potato starches, respectively. Similar trends were observed when three potato starches with different phosphorus contents were fortified with calcium [7]. Starch pasting properties appear to be important to predict the qualities of food products such as noodles and sauces. Divalent cations, such as calcium and magnesium, of potato starch with high starch-bound phosphate clearly play roles in starch pasting properties. Previous reports indicated distinct reductions in peak viscosity and breakdown due to the fortification of potato starch with calcium [7] and magnesium [18]. By comparison, the fortification of potato starch with potassium [7,18] and sodium [7] resulted in increases in peak viscosity and breakdown. Our present data for starch pasting properties, *i.e.*, peak viscosity and breakdown, were in agreement with the previous data mentioned above. DSC has been widely used to analyze starch gelatinization, but there has been limited information regarding the contribution of calcium and magnesium of potato starch to the gelatinization properties measured by DSC. According to the recent study of Fortuna *et al.* [18], in potato starch, the fortification of magnesium did not significantly influence the values of To and Tp, while it contributed to a slight reduction in ∆H. On the basis of the previous and present results, fortification of calcium or magnesium did not have a large impact on the gelatinization properties of potato starch. RS is defined as starch that avoids degradation by amylolytic enzymes in small intestine and behaves physiologically as a dietary fiber [24]. The determination of RS is based on the enzyme digestion of starch. Raw potato starch is well known to be difficult to digest by amylolytic enzymes, having a large amount of RS [2,6,25,26,27]. Supporting this, we demonstrated extremely high values of RS content (93.0% to 95.5%) of all potato starches examined in this study. Additionally, no significant change in RS content was detected due to calcium- and magnesium-fortification of the control potato starch. Contrary to this, Fortuna *et al.* [18] indicated that magnesium-fortified potato starch was more susceptible to enzyme digestion than was the control potato starch. Islam and Azemi [22], reporting on the effect of calcium fortification on enzymatic digestibility of native and hydroxypropyl rice starch, found that calcium fortification-induced amylolysis by pancreatic α-amylase yet somehow inhibited amyloglucosidase attack. The explanation for the contribution fortification with calcium and magnesium to starch digestibility is an open topic that requires further investigation.

The present study has revealed an effective way to prepare calcium- and magnesium-fortified potato starches with drastically decreased values of peak viscosity and breakdown using potato starch produced at a local factory in Hokkaido. The fortified potato starches have extremely large amounts of RS similar to the control starch. From the viewpoint of both potential health benefits and functional properties, calcium- and magnesium-fortified potato starches are value-added food materials. Our findings would expand the utilization of the potato starch produced in Hokkaido. Application of the fortified potato starches to food production is now in progress.

## 3. Experimental Section

### 3.1. Materials

Potato starch obtained from Toubu Tokachi Noukouren Starch Factory (Urahoro, Hokkaido, Japan) was used as the control potato starch in this study. Calcium chloride, anhydrous, (CaCl_2_) and magnesium chloride hexahydrate (MgCl_2_·6H_2_O) were purchased from Wako Pure Chemical Industries, Ltd. (Osaka, Japan). 

### 3.2. Preparation of Calcium- and Magnesium-Fortified Potato Starches

To prepare the calcium-fortified potato starch, the control potato starch (100 g) was suspended in a 0.005% to 1% CaCl_2_ solution (300 mL) and held for 3 h. The supernatant was discarded, and the remaining starch pellet was washed twice with distilled water, and then dried at 20 °C. To prepare the magnesium-fortified potato starch, control potato starch (100 g) was suspended in a 0.02% to 2% MgCl_2_·6H_2_O solution (300 mL) and held for 3 h. The supernatant was discarded, and the remaining starch pellet was washed twice with distilled water and then dried at 20 °C. The obtained starch samples were stored at 4 °C until analysis.

### 3.3. Determination of Starch Properties

For all starch samples examined, the RVA paste viscosity at 4% starch suspension (dry-weight basis, w/w) was determined as previously reported [17]. The calcium content of all calcium-fortified potato starch samples was determined. Similarly, the magnesium content of all magnesium-fortified potato starch samples was determined. For the representative calcium- and magnesium-fortified potato starches and the control potato starch, analysis of the content of five minerals—phosphorus, calcium, magnesium, sodium, and potassium—was performed. In addition, DSC analysis and the RS measurements were conducted using these starches. Phosphorus content was determined in accordance with Noda *et al.* [17], while the content of calcium, magnesium, sodium, and potassium was as previously reported [9]. DSC thermal properties at 30% starch suspension (dry-weight basis, w/w) were determined using a DSC 6100 (Seiko Instruments, Tokyo, Japan) in accordance with Noda *et al.* [17]. The RS content (the ratio of RS to total starch) was determined by the Megazyme Resistant Starch Assay Kit, 05/2008 (Megazyme International Ireland Ltd., Co., County Wicklow, Ireland) in accordance with the AOAC method 2002.02 [26]. Measurements of RVA paste viscosity and DSC thermal parameters were carried out in triplicate. RS determinations were replicated four times, while the estimations of minerals were performed only once. 

### 3.4. Statistical Analysis

The RVA paste viscosity averages were computed, and the Duncan’s multiple range tests were conducted to measure variations in the RVA paste viscosity among various concentrations of CaCl_2_ and among various concentrations of MgCl_2_ aqueous solutions. Additionally, DSC thermal parameters and RS content averages were computed, and the Duncan's multiple range tests were conducted to measure variations in the DSC thermal parameters and RS content among the representative calcium- and magnesium-fortified potato starches and the control potato starch. Significance was defined at *p* < 0.05.

## 4. Conclusions

Calcium- and magnesium-fortified potato starches were successfully prepared by immersion in CaCl_2_ and MgCl_2_ aqueous solutions, respectively. As a result, the potassium in the control potato starch was replaced by calcium and magnesium with these treatments. Calcium- and magnesium-fortified potato starches exhibited manifestly lower peak viscosity and breakdown than did the control potato starch. In contrast, gelatinization temperature and enthalpy of calcium- and magnesium-fortified potato starches were similar to those of the control potato starch. High RS content (around 94%) was observed in the calcium- and magnesium-fortified potato starches, and no significant difference in RS content was found as a result of calcium- and magnesium fortification of the control potato starch. Calcium- and magnesium-fortified potato starches appear to be important food sources for sources of calcium and magnesium, respectively. In addition, they possess good viscosity stability and high amounts of RS, which has received much attention for its health benefits. Our experimental results should provide useful information for the food industry to make better use of potato starch.
